# Hyperoxia Decreases Glycolytic Capacity, Glycolytic Reserve and Oxidative Phosphorylation in MLE-12 Cells and Inhibits Complex I and II Function, but Not Complex IV in Isolated Mouse Lung Mitochondria

**DOI:** 10.1371/journal.pone.0073358

**Published:** 2013-09-02

**Authors:** Kumuda C. Das

**Affiliations:** Department of Anesthesiology, Texas Tech University Health Sciences Center, Lubbock, Texas, United States of America; University of Mississippi, United States of America

## Abstract

High levels of oxygen (hyperoxia) are frequently used in critical care units and in conditions of respiratory insufficiencies in adults, as well as in infants. However, hyperoxia has been implicated in a number of pulmonary disorders including bronchopulmonary dysplasia (BPD) and adult respiratory distress syndrome (ARDS). Hyperoxia increases the generation of reactive oxygen species (ROS) in the mitochondria that could impair the function of the mitochondrial electron transport chain. We analyzed lung mitochondrial function in hyperoxia using the XF24 analyzer (extracellular flux) and optimized the assay for lung epithelial cells and mitochondria isolated from lungs of mice. Our data show that hyperoxia decreases basal oxygen consumption rate (OCR), spare respiratory capacity, maximal respiration and ATP turnover in MLE-12 cells. There was significant decrease in glycolytic capacity and glycolytic reserve in MLE-12 cells exposed to hyperoxia. Using mitochondria isolated from lungs of mice exposed to hyperoxia or normoxia we have shown that hyperoxia decreased the basal, state 3 and state3 μ (respiration in an uncoupled state) respirations. Further, using substrate or inhibitor of a specific complex we show that the OCR via complex I and II, but not complex IV was decreased, demonstrating that complexes I and II are specific targets of hyperoxia. Further, the activities of complex I (NADH dehydrogenase, NADH-DH) and complex II (succinate dehydrogenase, SDH) were decreased in hyperoxia, but the activity of complex IV (cytochrome oxidase, COX) remains unchanged. Taken together, our study show that hyperoxia impairs glycolytic and mitochondrial energy metabolism in in tact cells, as well as in lungs of mice by selectively inactivating components of electron transport system.

## Introduction

Supplemental oxygen therapy is a frequent modality in critical care units and is required for patients with compromised oxygen availability. In addition, high levels of oxygen (hyperoxia) are used in conjunction with anesthetics for protection against hypoxemia [1]. Further, hyperoxia is administered to premature newborn infants, which is believed to be a factor in the development of BPD [2]. Although supplemental oxygen therapy is clinically important, significant oxygen toxicity in the lung resulting from hyperoxia is a major impediment in the use of oxygen therapy [3]. Perturbed energy metabolism in hyperoxia is believed to cause impaired lung growth and repair during and following hyperoxia. For example, mitochondrial dysfunction in hyperoxia has been shown to cause alveolar developmental arrest in a mouse BPD model [4]. Thus, understanding the molecular mechanisms of lung energy metabolism in hyperoxia is important to developing an appropriate intervention strategy for supplemental oxygen therapy.

Oxidation of biological fuels is a critical source of energy required for efficient functioning of various organs, including the lung. The oxidation of fuels such as NADH, pyruvate, or succinate is accomplished via the mitochondrial electron transport chain (ETC). The passage of electrons in the ETC generates a proton gradient due to pumping of protons to the intermembrane space resulting in the production of ATP by ATP synthase [5]. However, electron leak during the passage of electrons via the ETC produces superoxide anions (O_2_^.−^), which are converted to various reactive species of oxygen (ROS), and are detrimental to the very ETC that produces them. ETC has been shown to generate higher levels of ROS in hyperoxia [6], [7]. Although enhanced mitochondrial ROS generation, decreased ETC activity and ATP production are major molecular factors in lung dysfunction in hyperoxia, little is known about the effect of hyperoxia on specific mitochondrial complexes. For example, mitochondrial studies have utilized the assay of activities of enzymes of complexes I–IV to understand the mitochondrial dysfunction, however, these studies may not reflect the real mitochondrial function or dysfunction due to the fact that moderate changes in the activities of these enzymes have little effect on overall system behavior [5].

In addition to oxidative phosphorylation, glycolysis is another energy yielding pathway in the cells. Both of these energy-yielding pathways dynamically shift due to change in nutrient environment and oxygen availability. For example, cells switch to glycolysis in the absence of oxygen. This metabolic adaptation allows the cells to survive, albeit at a minimal level of proliferative capacity such as hypoxic solid tumor cells. Hyperoxia has been shown to induce high glucose uptake in A549 cells [8]. Studies have shown that glucose utilization increases in hyperoxia as a compensatory mechanism due to disruption of oxidative phosphorylation [9], [10]. In contrast to the study of Allen and White [8], other studies have shown that although glycolysis is increased in hyperoxia there was no increase in the ATP production and cells eventually died [9], [10]. However, the effect of hyperoxia on glycolysis, glycolytic capacity or glycolytic reserve has not been adequately studied. It is unknown whether hyperoxia impairs the glycolytic reserve of cells or modulates the dynamic response of glycolysis. Using sequential addition of glucose, oligomycin or 2-D-glucose (2-DG) in the XF24 analyzer, it is possible to determine glycolysis, glycolytic reserve or glycolytic capacity of cells in normoxia or hyperoxia.

Mitochondrial function can be assessed in intact cells or isolated mitochondria with pros and cons for each method. While the cellular analysis is closer to the *in vivo* milieu of mitochondria, the effect of oxygen on substrate oxidation and inhibition of non-mitochondrial redox conditions is expected to affect cellular bioenergetics studies. On the other hand, isolated mitochondrial bioenergetics with stand-alone substrate utilization and specific functional aspect of each complex can be evaluated using specific substrates and inhibitors. But isolated mitochondria may not represent the *in vivo* conditions [5]. We have utilized both a cellular system and isolated mitochondria to determine how hyperoxia modulates energy production and substrate utilization in the lung mitochondria. We have utilized the Seahorse XF24 instrument to determine oxygen consumption in normoxia and hyperoxia in real-time, and have shown how the function of each complex is affected by specific use of substrates and inhibitors [11]. Our study shows that the mitochondrial ETC of alveolar epithelial type II pneumocytes of mouse origin (MLE-12) loose their spare respiratory capacity in addition to decreased rate of basal respiration. We also show that mitochondrial function is severely impaired in the lungs of mice exposed to hyperoxia. Although the function of complex I and II are decreased in hyperoxia the complex IV of mice lung mitochondria is remarkably resistant to dysfunction due to hyperoxia. We also determined the effect of hyperoxia on glycolytic capacity, glycolytic reserve and glycolysis using the XF24 mitochondrial analyzer. Our data show that, whereas the rate of glycolysis remained unchanged in hyperoxia, the glycolytic capacity and the glycolytic reserve of MLE-12 cells were significantly decreased.

## Materials and Methods

Antimycin A, oligomycin, carbonyl cyanide *p*-trifluoromethoxyphenylhydrazone (FCCP), rotenone, succinate, malate, pyruvate, ascorbate (Asc) and N,N,N′,N′ – Tetramethyl p-phenylenediamine (TMPD) were purchased from Sigma chemical company (St. Louis, MO). Other chemicals for mitochondrial isolation buffer and mitochondrial assay buffer were also purchased from Sigma Chemical Co. Mito Stress kit and Glycolytic Stress kit were purchased from Seahorse Biosciences, Billerica, MA. Oligomycin, rotenone, antimycin A and FCCP were prepared in ultrapure DMSO (Sigma Chemical Co., St. Louis, MO) at 2.5 mM. ADP, succinate, malate, pyruvate were prepared in ultrapure water and the pH was adjusted to 7.2 using KOH. Pyruvate was always freshly prepared [11]. Oligomycin, glucose and 2-D-glucose (2-DG) were prepared following manufacturer's instructions that were supplied in the XF glycolysis test kit (Seahorse Bioscience, Billerica, MA).

### MLE-12 cells and exposure to hyperoxia

MLE-12, mouse lung epithelial type II cells were purchased from ATCC (Manassas, VA). Cells were cultured in HITES (Hydrocortisone, Insulin, Transferrin, Estrogen) media[12] [RPMI1640, 2% FBS, insulin(5 μg/mL), transferrin(10 μg/mL), sodium selenite (30 nM), hydrocortisone (10 nM), β-estradiol (10 nM), HEPES (10 nM)]. Cells were seeded at 100,000 cells/well in the 24-well V7 XF assay plate (Seahorse Bioscience, Billerica, MA). After 5 hours of seeding cells were exposed to 24 h hyperoxia (95% oxygen +5% CO_2_) or normoxia (room air) containing 1 mL of media. Cells were mixed with 1% Trypan blue and viable cell number was counted using a hemocytometer. Before the assay cell media was aspirated and replaced with 1X XF assay media without serum, antibiotics or Sodium bicarbonate (Seahorse Bioscience, Billerica, MA), but with 1 mM pyruvate and 25 mM glucose.

### Animals, exposure to hyperoxia and isolation of lung mitochondria

Wildtype (WT) C57BL/6 mice were purchased from Jackson laboratory and were bred and maintained in the animal facility of Texas Tech University Health Sciences Center (TTUHSC). The protocol was approved by the Institutional Animal Care and Use committee (IACUC) of the TTUHSC. Mice were exposed to room air (normoxia) or hyperoxia (90% oxygen) for 48 hours in Plexiglas animal exposure chamber (Biosperix, NY). The oxygen concentration was continuously monitored using Proox110-O_2_ and the CO_2_ level was monitored with Proox-CO_2_ (Biospherix Co, NY). The chamber contained soda lime in a container for removal of excess CO_2_. Lungs of mice (6–8 weeks old male or female) were surgically removed from anesthetized animals. Mitochondria from lungs were isolated according to Reiss [13] with modifications. Briefly, lungs were perfused with cold 1X PBS and minced in buffer A (0.32 M sucrose, 1 mM EGTA, 10 mM Tris-HCl, pH 7.4 and 0.2% lipid free BSA) using sharp scissors. Minced lung was homogenized with a Potter Elvehjem homogenizer (2 strokes). The Potter Elvehjem homogenized lung was further homogenized in a glass dounce homogenizer at 1:10 w/v in buffer A. The homogenate was centrifuged at 500×g for 10 minutes followed by the collection of the supernatant. The supernatant was centrifuged at 1600×g in a volume of 10 ml. The supernatant was passed through a 30 μm filter and further centrifuged at 12,500×g for 10 minutes. The resulting pellet was suspended in 2 ml buffer and transferred to a glass dounce homogenizer and subjected to 2–3 strokes. To the 2 ml of suspension 18 ml buffer A was added and the resulting 20 ml suspension was centrifuged at 25,000×g for 5 minutes. The mitochondrial pellet was washed in 2 ml buffer A without BSA and centrifuged at 22,000×g for 5 minutes. The pellet was resuspended in 300 μl of buffer A without BSA. Mitochondrial protein was quantitated using Bradford assay (Bio-Rad, CA). Mitochondria were suspended in 1X mitochondrial assay buffer (MAS); 70 mM sucrose, 220 mM mannitol, 10 mM KH_2_PO_4_, 5 mM MgCl_2_, 2 mM HEPES, 1 mM EGTA and 0.2% fatty-acid free BSA. The mitochondrial suspension (10 μg/50 μl volume) was aliquoted to each well of V7 cell culture plate (Seahorse Bioscience, Billerica, MA), and the plate was centrifuged in swinging bucket rotor with microplate rotor adaptor (Eppendorf 5810R with microplate adaptor, Eppendorf, Germany) for 20 minutes at 2000 rpm at 4°C to allow isolated mitochondria to attach to the bottom of the plate [11].

### MLE-12 Bioenergetics using the XF24 Flux Analyzer

MLE-12 cells were seeded at 100,000 cells/well of XF24 cell plate (Seahorse Bioscience, Billerica, MA) 30 hours before the assay. Optimization of reagents was performed using the Mito stress test kit from Seahorse Bioscience (Billerica, MA) using the protocol and algorithm program in the XF24 analyzer. Briefly, the concentration of oligomycin, FCCP, antimycin A and rotenone were optimized over a concentration range using 100,000 MLE-12 cells seeded onto each well. The bioenergetics assay was run in one plate with 10–20 replicates. The assay was repeated at least 3 times using 3 v7 plates for normoxia and 3 plates for hyperoxia. The following indices of mitochondrial respiration were calculated based on Brand and Nicholls [5]. Basal OCR is [OCR with substrates− OCR with rotenone and antimycin A]. ATP turnover is the OCR used for mitochondrial ATP synthesis, and is the OCR due to oligomycin. However, ATP could be generated in the glycolysis and oligomycin does not inhibit that. Therefore, here we use the term “ATP turnover” strictly for ATP generated via oxidative phosphorylation due to ATP synthase. Maximal OCR is [OCR with FCCP− OCR with rotenone and antimycin A. The spare respiratory capacity (SRC) is OCR with FCCP – Basal OCR].

### Assay for glycolysis, glycolytic capacity and glycolytic reserve in MLE-12 cells

MLE-12 cells were seeded using HITE's media at 70,000 cells/well of XF24 cell plate (Seahorse Bioscience, Billerica, MA) 24 hours before the assay. On the day of the assay, the media was changed to DMEM (without serum, glucose or bicarbonate, but with 2 mM Glutamine), and incubated for 1 hour before the assay in a non-CO_2_ incubator at 37°C. Injections of glucose (10 mM final), oligomycin (3 μM final) and 2-DG (0.1 M final) were diluted in the DMEM media and loaded onto ports A, B and C respectively. The machine was calibrated and the assay was performed using glycolytic stress test assay protocol as suggested by the manufacturer (Seahorse Bioscience, Billerica, MA). The assay was run in one plate with 10–20 replicates. The assay was repeated at least 3 times using 3 v7 plates for normoxia and 3 plates for hyperoxia. The rate of glycolysis is the ECAR after the addition of glucose. Glycolytic capacity is the rate of increase in ECAR after the injection of oligomycin following glucose. Oligomycin inhibits mitochondrial ATP production and therefore shifts the energy production to glycolysis with increase in ECAR revealing maximum glycolytic capacity of the cells. The glycolytic reserve is the difference between glycolytic capacity and glycolysis rate.

### Bioenergetics of isolated lung mitochondria

The XF24 instrument was equilibriated at 37°C overnight and the measure and mix cycles were made similar to the published report for rat heart mitochondria [5], [11]. Ten micrograms of mouse lung mitochondria was plated in each well of the V7 culture plate in a volume of 50 μl containing 1X MAS with 10 mM succinate and 2 μM rotenone as substrate for the coupling assay and 10 mM pyruvate, 2 mM malate and 4 μM FCCP was added to 1X MAS for the electron flow experiment. The XF plate was centrifuged with substrate containing mitochondrial homogenate for 20 min at 2000 rpm in a swinging bucket rotor using plates to hold the XF plate. After centrifugation, 450 μl of substrate containing 1X MAS was added to each well, and the plate was incubated at 37°C in a non-CO_2_ incubator for 8–10 minutes [11]. While the plate was centrifuged, the XF cartridge was prepared for injection for ports A, B, C and D. ADP (50 μl), oligomycin (55 μl), FCCP (60 μl) and antimycin A (65 μl) were loaded onto ports A, B, C, D respectively. The final concentrations of injections were 4 mM ADP, 2.5 μM oligomycin, 4 μM FCCP and 4 μM antimycin A. The injections for electron flow experiment was prepared as follows: Port A, 20 μM rotenone (50 μl), Port B, 100 mM succinate (55 μl), Port C, 40 μM antimycin A (60 μl), port D, 10 mM ascorbate with 1 mM TMPD (65 μl). The cartridge was calibrated by the machine, and following calibration the XF plate with mitochondria attached to the bottom was introduced to the machine and the assay continued using protocol developed by Rodgers et al for rat heart [11]. The coupling and electron flow assay for normoxia and hyperoxia were run in one v7 assay plate with 5 replicate wells for each assay. This was repeated at least 3 times with 3 animals for normoxia and 3 animals for hyperoxia.

### Enzymatic assay of NADH dehydrogenase, Succinate dehydrogenase and Cytochrome c oxidase

Mice were exposed to normoxia or 48 hours hyperoxia following which lungs were removed and mitochondria were isolated as mentioned earlier. Mitochondria were briefly sonicated in mitochondrial isolation buffer (50 mM Tris-HCl, pH 7.4, 2 mM EDTA, 5 mM MgCl_2_ and 0.25 M sucrose). Protein was determined by Bradford assay (Bio-Rad, CA) and equal amount of mitochondrial protein was used for each assay. NADH-dehydrogenase activity was measured as rotenone-sensitive reductase by the rate of change of absorbance of reduced NADH in presence of cytochrome c according to publish methods with modifications [10]. SDH activity was determined by the published method of Veeger et al. with modifications [10], [14]. Cytochrome c oxidase activity was measured by published method [15]–[17].

### Statistical Analysis

The statistical evaluations were performed with analysis of variance (ANOVA) in Graph Pad Prism software. ANOVA was used for analysis of more than 3 means. Where necessary post-test was performed with Fisher's LSD test following ANOVA. The minimum number of n = 3 was employed in all experiments performed with 5–20 replicate wells in the Seahorse XF24 analyzer. For animal experiments, n = 3 was used for normoxia or hyperoxia. Mitochondria from one normoxic lung and one hyperoxic lung were assayed in a single microplate with 5 replicates wells for each of the coupling and the electron flow experiment. The experiment was repeated at least 3 times with 3 animals. Enzyme activities were compared between normoxia and hyperoxia using t-test. The results were considered significant at p<0.05.

## Results

### Optimization of XF assay for MLE-12 cells

**Figure 1:**
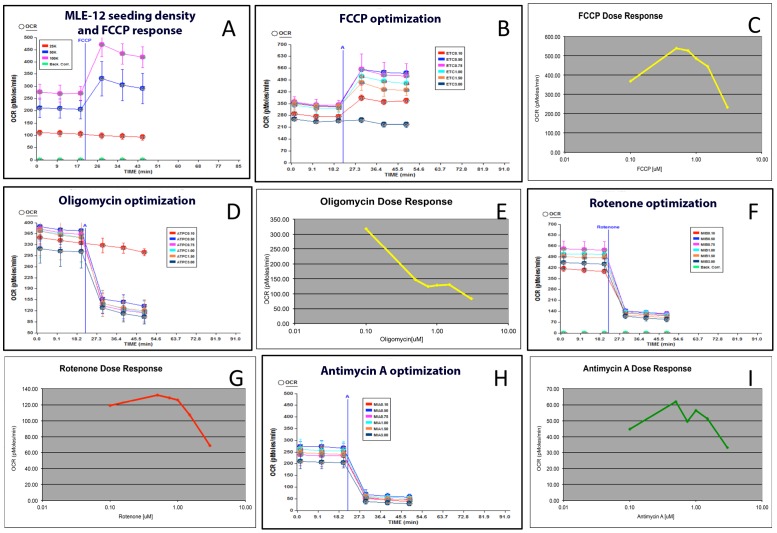
Optimization of MLE-12 cell seeding and optimization of compounds used in mitochondrial bioenergetics assay in the XF24 flux analyzer. (**A**) The effect of seeding density on OCR. MLE-12 cells were seeded at 25,000, 50,000 or 100,000 cells/well and after 16 hours the basal OCR and OCR due to FCCP was determined using XF24 Flux analyzer as described in the experimental procedure; (**B & C**) Optimization of FCCP using Mito Stress Test kit (Seahorse Bioscience, Billerica, MA). The range of final concentrations tested was 3, 1.5, 1.0, 0.75, 0.5 and 0.1 µM. Each concentration had 4 replicates in the V7 micro-well plate of XF24; (**D & E**) Optimization of oligomycin; (**F & G**) Optimization of rotenone and (**H & I**) optimization of antimycin A.

We optimized the seeding density and concentrations of the injection compounds before performing the bioenergetics assay. The OCR of MLE-12 cells was increased in a linear fashion when 25,000, 50,000 or 100,000 cells/well were seeded in V7 the microplate (Seahorse Bioscience; Fig 1A). Whereas 50,000 or 100,000 cells/well responded to FCCP resulting in maximal respiration rate, 25000 cells/well did not show any increase due to FCCP treatment (Fig 1A). We tested a range of FCCP concentrations (3, 1.5, 1.0, 0.75, 0.5 and 0.1 μM) for maximal OCR determination (Fig 1B). The optimal concentration of FCCP was found to be 0. 5 μM (Fig 1C). Increasing the concentration of FCCP beyond 0. 5 μM did not increase the OCR (Fig 1C). The optimal concentration for oligomycin was determined to be 3 μM (Fig 1D & E). Likewise, the optimal concentration of rotenone and antimycin A was found to be 3 μM each (Fig 1F & G & H & I). We used 100,000 cells/well for initial seeding and optimal concentration of reagents for all subsequent experiments.

### Effect of hyperoxia on MLE-12 bioenergetics

**Figure 2:**
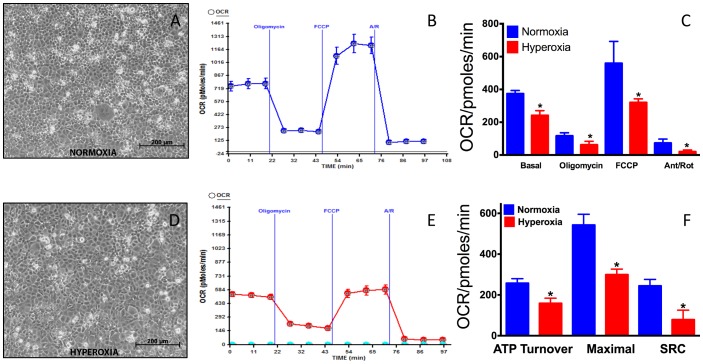
Mitochondrial bioenergetics of MLE-12 cells in normoxia and hyperoxia. MLE-12 cell were seeded in V7 cell plate at a density of 100,000 cells/well. After 5 hours one micro titer plate was exposed hyperoxia (95% O_2_+5% CO_2_) for 24 hours and the other to normoxia. Mitochondrial bioenergetics assay was performed as described in the methods using XF24 instrument. (**A**) Morphology of MLE-12 cells in normoxia; (**B**) A representative graph of OCR output from XF24 analyzer of MLE-12 cells and its response to oligomycin, FCCP, rotenone/antimycin A in normoxia; (**C**) Graph of basal OCR and OCR in response to oligomycin, FCCP and antimycin A/Rotenone in normoxia and hyperoxia. (**D**) Morphology of MLE-12 cells in 24 h hyperoxia in the micro-well; (**E**) A representative graph of OCR output from XF24 analyzer of MLE-12 cells and its response to oligomycin, FCCP, rotenone/antimycin A in 24 h hyperoxia; (**F**) The effect of hyperoxia on ATP turnover, maximal respiration and spare respiratory capacity (SRC), computed as described in the methods. Triplicate samples (n = 3) with 5 replicate for each well per group per experiment. The data are normalized to number of cells (100,000 cells) on the plate after each assay as the growth of cells might have altered in normoxia or hyperoxia. *Significant at p<0.05, Analysis of variance (ANOVA) followed by Fisher's LSD multiple comparison (Graph Pad Prism v6).

We exposed MLE-12 cells to 24 hours of hyperoxia and determined the bioenergetics profile of cells. The morphological features of MLE-12 cells (seeded at 100,000 cells/well) is presented in Fig 2A (normoxia) and Fig 2D (24 h hyperoxia). As seen in Fig 2A the normoxic cells are tightly packed and appears to be smaller in size. However, MLE-12 cells exposed to hyperoxia (Fig 2D) are larger in size and cover entire surface of micro-well. The hyperoxia exposed plate had an average of 154937±1159 cells/well compared to 195750±2386 cells/well in the normoxia plate, suggesting significant growth inhibition of cells (p<0.0001, t-test). Hyperoxia is known to cause growth arrest of cells [18], [19]. Consequently, all MLE-12 OCR and ECAR data have been normalized per 100,000 viable cells. As demonstrated in Fig 2B, C & E, MLE-12 cells exposed to hyperoxia showed significant decrease in basal OCR compared to normoxia. Injection of oligomycin, an ATP synthase inhibitor decreased the OCR due to inhibition of oxidative phosphorylation, which was further decreased in hyperoxia (Fig 2C & E). Further, injection of FCCP, a mobile proton carrier (uncoupler) increased the OCR in normoxia. However, FCCP-mediated increase in OCR was decreased in hyperoxia. Finally, the mitochondrial respiration was inhibited using complex I and III inhibitors rotenone and antimycin A, demonstrating that the oxygen consumption was due to mitochondrial respiration. We determined the effect of hyperoxia on the ATP turnover, maximal respiration and the spare respiratory capacity (SRC) as mentioned in the experimental procedure. Our data (Fig 2F) demonstrate that ATP turnover, maximal respiration and SRC were significantly decreased in hyperoxia compared to normoxia.

### Effect of hyperoxia on glycolysis, glycolytic capacity and glycolytic reserve of MLE-12 cells exposed to normoxia or hyperoxia

**Figure 3:**
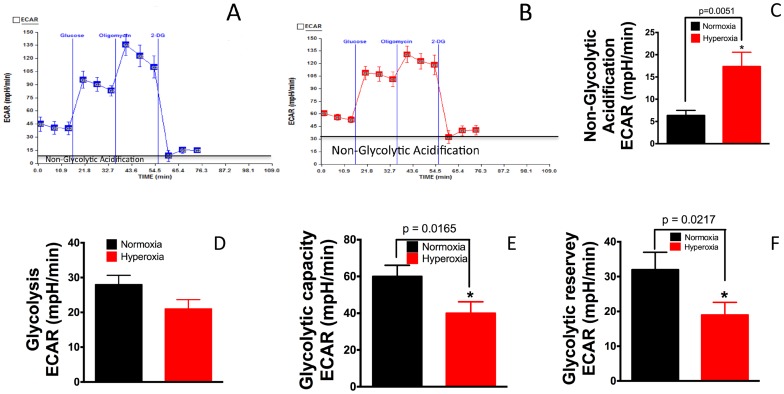
Glycolysis, glycolytic capacity and glycolytic reserve of MLE-12 cells in normoxia and hyperoxia. MLE-12 cell were seeded in V7 cell plate at a density of 70,000 cells/well and exposed to normoxia or hyperoxia (95% O_2_+5% CO_2_) for 24 hours. Glycolysis assay was performed using glycolysis test kit from Seahorse Biosciences according to manufacture's protocol using XF24 instrument. (**A**) A representative graph output from XF24 showing the ECAR response to glucose, oligomycin and 2-DG in normoxia; (**B**) A representative graph output from XF24 showing the ECAR response to glucose, oligomycin and 2-DG in 24 hours hyperoxia; (**C**) Non-glycolytic acidification, (**D**) Glycolysis (**E**) Glycolytic capacity; (**F**) Glycolytic reserve. The minimum number of n = 3 with 10–20 replicate wells per group was employed for all experiments. Data normalized to 100,000 cells; *Significant at p<0.05; Student t-test (Graph Pad Prism v6).

We sought to determine whether hyperoxia induces glycolytic pathway of energy production due to impairment of oxidative phosphorylation. As shown in Fig 3 the non-glycolytic acidification of MLE-12 cells was significantly increased in hyperoxia when no glucose, but glutamine was present in the media (Fig 3C). The ECAR was significantly increased in MLE-12 cells when glucose was injected to wells, demonstrating that high glucose induces glycolysis (Fig 3A, B & D). However, there was no significant difference in glycolysis in cells exposed to 24 h hyperoxia or normoxia (Fig 3D). It has been shown that glucose utilization is increased in hyperoxia, but the cells die due to loss of ATP production [9], [10]. Therefore, we examined the maximal glycolytic capacity of cells exposed to normoxia or hyperoxia using oligomycin to inhibit oxidative phosphorylation. As shown in Fig 3E the glycolytic capacity was significantly decreased in hyperoxia compared to normoxia. The glycolytic reserve which is the difference between glycolytic capacity and glycolysis was also significantly decreased in hyperoxia (Fig 3F).

### Optimization of isolated mouse lung mitochondria for bioenergetics using XF analyzer

**Figure 4:**
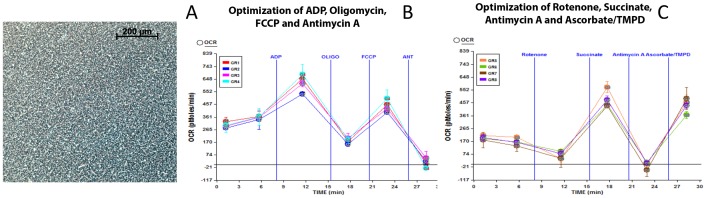
Isolated mouse lung mitochondria and compound optimization for XF analysis. (**A**) Adherence of 10 μg of mouse lung mitochondrial protein in XF24 cell plate Following XF assay. Mouse lung mitochondria was isolated and attached to XF24 microplate as described in the methods; (**B**) **Compound titration for XF coupling assay in isolated lung mitochondria.** GR1, 3 μM FCCP, 2.0 μg oligomycin and 3 mM ADP; GR2, 4 μM FCCP, 3.0 μg oligomycin and 4 mM ADP; GR3, 6 μM FCCP, 4.0 μg oligomycin and 5 mM ADP; GR4, 8 μM FCCP, 5.0 μg oligomycin and 6 mM ADP. (**C**) **Compound titration for XF electron flow assay in isolated lung mitochondria.** GR5, 2 µM rotenone, 5 mM succinate and 5 mM ascorbate/1 mM TMPD); GR6, 3 μM rotenone, 10 mM succinate and 10 mM ascorbate/1 mM TMPD); GR7, 4 μM rotenone, 15 mM succinate and 15 mM ascorbate/1 mM TMPD); GR8, 5 μM rotenone, 20 mM succinate and 20 mM ascorbate/1 mM TMPD). Three replicate wells are run in the same plate for each concentration of compounds.

We sought to determine the response of lung mitochondria to hyperoxia using XF analyzer, because the response of intact cells to hyperoxia could be different from animals exposed to hyperoxia. First, we optimized the amount of mitochondrial protein, and the concentrations of oligomycin, FCCP, antimycin A and rotenone on the rate of respiration for the XF assay since this is the first report of XF assay of mouse lung mitochondria. As demonstrated in Fig 4A, 10 μg mitochondrial protein could be attached to the XF microplate without any lifting. We used 10 μg of mitochondrial protein to optimize ADP (3 to 8 mM), FCCP (3 to 8 μM) or oligomycin (2 to 5 μg). The amount of antimycin A was kept constant at 4 μM. As shown in Fig 4B, there was no significant change in OCR response to various concentration of compounds used in the assay. Similarly, in the electron flow experiment we used rotenone (1 to 4 μM), succinate, (5-20 mM) or Asc/TMPD (5 to 20 mM) to determine the optimal level of these compounds, but there was no significant difference among these concentrations for a specific compound (Fig 4C). Thus, our final concentrations were 4 mM ADP, 4 μM FCCP, 2.5 μM oligomycin and 4 μM antimycin A for coupling assay. For the electron flow experiment the final concentrations were 2 μM rotenone, 10 mM succinate, 4 μM antimycin A, and 10 mM ascorbate with 1 mM TMPD. The coupling experiment (rates of state2, state3, state4_O_ and state3 μ) was performed using succinate as substrate and using oligomycin as a coupling agent. FCCP was used as an uncoupling agent to evaluate the effect of hyperoxia on the maximal respiration rate. Rotenone and antimycin A were used to inhibit complex I and complex III respirations. The flow of electrons via each complex (I–IV) was determined using specific substrates, pyruvate and malate for complex I, succinate for complex II and Ascorbate/TMPD for complex IV.

#### Coupling

**Figure 5:**
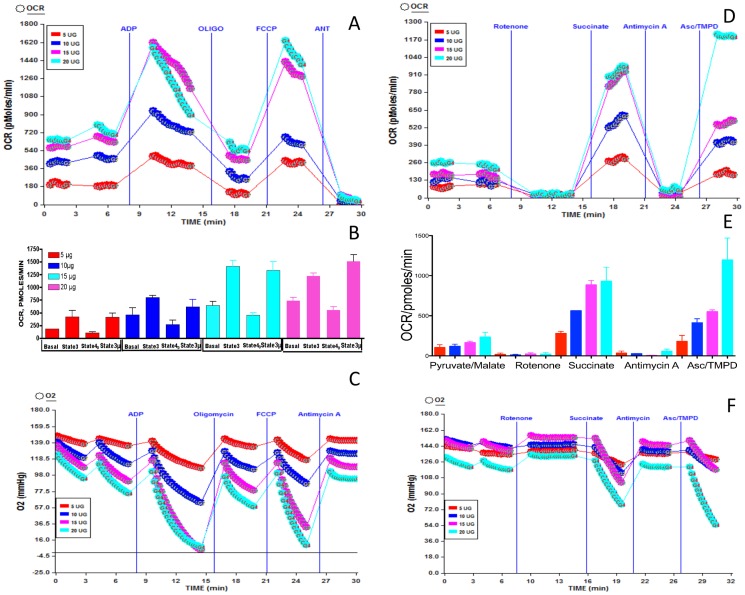
Optimization of lung mitochondrial protein for bioenergetics analysis using XF24. Mouse lung mitochondria were isolated as described in the methods. 5, 10, 15 or 20 μg of lung mitochondrial protein was attached to microplate as described in the methods and mitochondrial coupling and electron flow assays were performed as described. (**A**) Coupling assay using isolated lung mitochondrial protein and OCR response to ADP, Oligomycin, FCCP and antimycin A; (**B**) Basal, state3, state4_O_ and state3 μ respirations of 5, 10, 15 or 20 μg lung mitochondrial protein; (**C**) Absolute oxygen concentration in the micro-chamber with respect to amounts of mitochondrial protein; (**D**) Electron flow experiment in isolated lung mitochondrial protein (5, 10, 15 or 20 μg mitochondrial protein/well); (**E**) Graph of OCR data in electron flow experiment and OCR response to rotenone, succinate, antimycin A or Asc/TMPD; (**F**) Absolute oxygen concentration in the micro-chamber with respect to amounts of mitochondrial protein. Two or three replicate wells were used for each mitochondrial protein concentration for the optimization assay.

This is the process where the flow of electrons generates proton motive force (pmf) due to extrusion of protons. In the presence of ADP, the inward flow of protons via ATP synthase produces energy in the form of ATP. As an initial evaluation we used 5, 10, 15 or 20 μg of lung mitochondrial protein for coupling experiment. The basal OCR was increased in a linear manner demonstrating that the rate of respiration is directly related to amount of isolated mitochondria (Fig 5A & B). However, there was no significant difference in the OCR between 15 or 20 μg mitochondrial protein demonstrating saturation levels of mitochondria per well (Fig 5A&B). As shown in Fig 5B, the basal, state3, state4_O_ and state3 μ OCR were linearly increased when 5–15 μg mitochondrial protein was used/well. However, the OCR did not increase in 20 μg mitochondrial protein/well indicating that a saturation level has reached. Additionally, at higher concentration of mitochondrial protein the absolute oxygen levels almost decreased to zero in the micro-chamber of the well, which could compromise the availability of oxygen for the subsequent measurement cycle (Fig 5C). The OCR was decreased with the use of oligomycin and increased with use of uncoupling agent FCCP demonstrating integrity of isolated lung mitochondria.

#### Electron Flow

In this experiment the flow of electrons from complex I to complex IV is monitored in the presence of FCCP, an uncoupler that carries protons away through the inner membrane without formation of a proton gradient. Therefore, ATP is not produced in this system. But the defects in a specific complex could be determined by decrease or increase in oxygen consumption when a complex-specific substrate is provided. We used pyruvate (10 mM) and malate (2 mM) in presence of uncoupling agent FCCP (4 μM) for allowing complex I respiration in an uncoupled state. Complex I respiration increased when 5 to 15 μg of mitochondrial protein was used per well. However, increasing the mitochondrial protein to 20 µg/well did not increase the OCR from that of 15 μg/well (Fig 5D & E), demonstrating saturation of mitochondria in the micro-well, although absolute oxygen concentration was not limiting (Fig 5F). Addition of rotenone stopped the complex I respiration as expected (Fig 5D&E). Injection of succinate induced the lung mitochondrial respiration via complex II. Although there was a linear increase in complex II respiration in 5–15 μg mitochondrial protein per well, plating 20 μg mitochondrial protein did not increase the OCR over that of 15 μg. Inhibition of complex III by antimycin A inhibited respiration as expected (Fig 5D & E). When Asc/TMPD was injected as electron donor to complex IV (Cytochrome oxidase) the OCR increased with concomitant drop in absolute level of oxygen (Fig 5D, E &F) in response to 5–20 μg mitochondrial protein with 20 μg mitochondrial protein showing very high OCR unlike OCR in response to other complexes. The absolute oxygen concentrations in the micro-well remained adequate in 20 μg mitochondrial protein (Fig 5F).

### Effect of hyperoxia on mitochondrial bioenergetics of mouse lung

To determine the effect of hyperoxia on mitochondrial bioenergetics we performed coupling and electron flow assays in mitochondria isolated from lungs of mice exposed to normoxia or hyperoxia.

#### Mitochondrial coupling in hyperoxic lung

**Figure 6:**
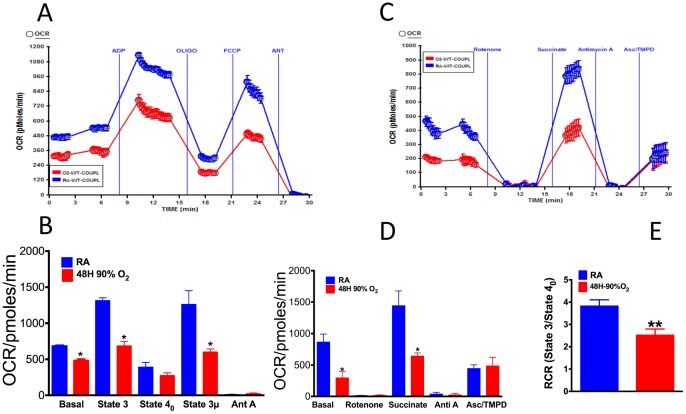
Effect of hyperoxia on mitochondrial bioenergetics of mouse lung. C57BL/6 mice were exposed to room air (normoxia, n = 3) or hyperoxia (90% oxygen, n = 3) for 48 hours. Following exposure lung mitochondria were isolated as described in the methods and analyzed in XF24 analyzer. (**A**) A representative graph output of coupling experiment of isolated lung mitochondrial protein in normoxia or hyperoxia; (**B**) Basal, state 3, state 4_0_ and state 3 μ respiration of isolated lung mitochondrial protein in normoxia or hyperoxia; (**C**) A representative graph output of electron flow experiment of mitochondria isolated form mice lung exposed to normoxia or hyperoxia; (**D**) OCR response of rotenone, succinate, antimycin A and ascorbate/TMPD. The experiments were run in 5 replicate wells for each group for coupling or flow. Statistical analysis was performed using ANOVA using Graph Pad Prism software. *Significantly different at p<0.05. (**E**) Graph of RCR of lung mitochondria in normoxia or hyperoxia; n = 3, student t-test, *Significant at p<0.05.

The effect of hyperoxia on basal respiration (state 2), phosphorylating respiration in presence of ADP (state 3), resting respirations in presence of oligomycin (state 4_o_), maximal respiration in presence of FCCP (state 3 μ), and the response to antimycin A was determined using 10 μg mitochondrial protein isolated from lungs of WT mice exposed to room air (RA) or hyperoxia. In the coupling assay, succinate (a FADH_2_-linked substrate of complex II) was used with rotenone to allow the mitochondria respire via complex II, as succinate without rotenone would form oxaloacetate from malate by the action of malate dehydrogenase. Oxaloacetate is impermeable to mitochondrial inner membrane and would accumulate and function as a potent inhibitor of succinate dehydrogenase, the complex II enzyme. As shown in Fig 6A and B hyperoxia significantly decreased complex II driven state 2 (basal) respiration in isolated lung mitochondria. State 3 respiration in the presence of ADP was significantly increased in mitochondria isolated from RA exposed mice, but it was decreased in mitochondria isolated from lungs of mice exposed to 48 hours of hyperoxia (Fig 6A & B). Likewise, the maximal respiration in response to uncoupler FCCP (state 3 μ) was decreased in hyperoxia-exposed lung compared to normoxia. Treatment of antimycin A decreased the respiratory rate as expected. We also determined the RCR (State 3/State 4o), which is an indicator of mitochondrial integrity. Our data (Fig 6E) show that RCR decreased in hyperoxia (2.571±0.160) compared to normoxia (3.824±0.162) demonstrating a mitochondrial dysfunction in hyperoxia.

#### Mitochondrial electron flow in hyperoxic lung

The electron flow experiment was performed using pyruvate and malate as substrates, which are oxidized via complex I. Exposure to hyperoxia decreased the basal OCR demonstrating a complex I malfunction in the isolated lung mitochondria (Fig 6C & D). Because the substrate was provided to the mitochondria isolated from hyperoxia or normoxia exposed mice, the data shows specific dysfunction of complex I to utilize the substrate, but not the substrate oxidation resulting in decreased OCR. The OCR was significantly increased in normoxia-exposed mitochondria in response to injection of succinate, which is oxidized via complex II, demonstrating a complex II driven respiration (Fig 6C & D). However, hyperoxia significantly decreased the OCR suggestive of complex II malfunction as succinate was supplied in vitro to the mitochondria. Inhibition of complex III by antimycin A was similar for normoxic or hyperoxic lung. We used ascorbate/TMPD, which is a direct electron donor for complex IV to determine whether complex IV function is compromised in hyperoxia similar to complex I or complex II. To our surprise the OCR following ascorbate/TMPD injection was similar in normoxic or hyperoxic lung mitochondria demonstrating that the complex IV function remains unaltered in response to hyperoxia (Fig 6C & D).

### Effect of hyperoxia on the activities of enzymes of complex I, II and IV

**Figure 7:**
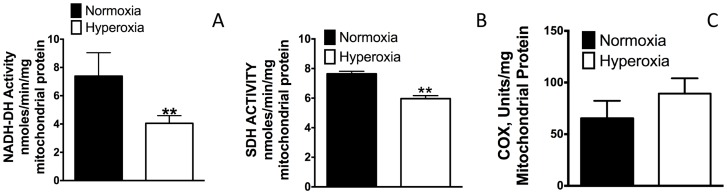
Effect of hyperoxia on NADH-DH, SDH and cytochrome c oxidase. Mice were exposed to normoxia or hyperoxia (48 h) following which mitochondria were isolated from lungs of mice as described in the methods section. Enzymatic assays were performed as described in the methods, (**A**) NADH-DH, (**B**) SDH (**C**) COX. A minimum of n = 3 employed for each enzyme assay. Student t-test is used to compare means. *Significant at p<0.05 level.

Our data in the flux analyzer show that OCR by a specific complex is affected in hyperoxia when other complexes are either inhibited or complex-specific substrate is provided in the assay. We determined the activity of a specific complex enzyme, which is a major constituent of the complex. For example, NADH-dehydrogenase represents complex I function and SDH and COX represent complex II and complex IV function. As shown in Fig 7A the activity of NADH-DH was decreased in hyperoxia compared to normoxia, which is consistent with our OCR data presented in Fig 6. The activity of SDH was decreased in hyperoxia compared to normoxia (Fig 7B), this is consistent with complex II OCR shown in Fig 6. The activity of cytochrome oxidase the terminal oxidase in complex IV remained unchanged in hyperoxia consistent with the OCR data. These studies further confirm our findings and show that OCR data could represent the functional modulation of a specific complex in the ETC chain if the substrate and inhibitors could be used following proper strategy.

## Discussion

In the present report we have shown that exposure of type II mouse lung epithelial cells (MLE-12) to 24-hours of hyperoxia decreases basal OCR, ATP turnover, maximal respiration and SRC. We have also shown that, whereas there was no significant change in the rate of glycolysis in normoxia or hyperoxia, glycolytic capacity and glycolytic reserve were significantly decreased in cells exposed to hyperoxia. In addition, mice exposed to 48 hours of hyperoxia show significant disruption of lung mitochondrial function primarily due to dysfunction of both complexes I and II. However, the function of complex IV remains unaltered in the lung mitochondria in mice exposed to 48 h hyperoxia. Basal, state 3 and state 3 μ respirations are decreased in hyperoxia compared to normoxia. Taken together, our data demonstrate that mitochondrial energy metabolism is significantly decreased in hyperoxia leading to decreased energy production in intact cells, as well as in the lungs of mice.

We have optimized mouse lung mitochondrial protein for analysis of mitochondrial function using the XF24 analyzer in the current investigation, as this is the first report of an XF assay using mouse lung mitochondria. A minimal amount of 10 μg lung mitochondrial protein is required for a single well for bioenergetics analysis in contrast to 200 or 300 micrograms required for analysis in the Clark-type electrode. Our data show that the isolated mitochondria are intact and not uncoupled due to the isolation procedure. The state 3 respiration in presence of ADP was similar to that of mitochondria isolated from either heart or liver [11]. To the best of our knowledge, we report for the first time the RCR values of mouse lung mitochondria to be 3.8 using succinate as substrate. We are not aware of any previous investigation that has reported mouse lung RCR values using the XF24 instrument. Our reported values are somewhat lower than a previously published report for rat lung mitochondria using a Clarke type electrode [20]. We believe that this could be due to species difference and the use of a Clarke-type oxygen electrode, where significant back-diffusion of oxygen from a Teflon stirrer bar or from the surrounding vessel could underestimate the state 4 respiration and thus result in an erroneously high RCR [5]. The functional analysis of our lung mitochondria remains solidly interpretable and the procedure offers rapid isolation and analysis of mitochondrial function using the XF24 assay system. Functional analysis of individual ETC complexes is possible using specific electron donors for each complex using the electron flow experiment. We used pyruvate/malate as substrates for the lung mitochondria in this report. However, other substrates such as glutamate/malate could be used or other substrates may be substituted; but the assay must be optimized for each substrate or electron donor. Our experiments (coupling and electron flow) were performed in a single XF24 plate with 5 replicate wells for each experiment that limits variations in the coupling and electron flow assays.

Lungs are direct target organs for oxygen, and high levels of oxygen are frequently used in critical care units, underscoring the relevance of mitochondrial function analysis in hyperoxia. Increased levels of ROS are known to be generated in the mitochondria in response to hyperoxia [21]. The passage of electrons in the ETC is increased in hyperoxia with concomitant production of higher levels of superoxide anion and other reactive oxygen species [7], [22]. Higher levels of ROS in the mitochondria could inactivate the components of the ETC resulting the impairment of energy production in hyperoxia. We evaluated the effect of hyperoxia on mitochondrial bioenergetics of mouse lung. The basal, state 3, state 4_0_ and state 3 μ respiratory rates are decreased in hyperoxia-exposed lung mitochondria. The activities of complex I and II were decreased in response to hyperoxia. Complex I is the major site where maximal amount of ROS are generated [23]. When pyruvate and malate, which are substrates for complex I, are used a decrease in complex I driven OCR in hyperoxia was noted (Fig 5C &D). Additionally, the complex II driven respiration due to addition of succinate was decreased in hyperoxia, demonstrating a dysfunction of complex II in addition to complex I. Our data show that the function of complex IV remained unchanged in hyperoxia. Direct electron donors to complex IV (Ascorbate/TMPD) showed that the complex function is similar to normoxic lung. Complex IV does not produce ROS [21], which transfers electrons finally to oxygen to produce water. Therefore, it is likely that superoxide generated during hyperoxia [23] results in damage to complex I and II. Because complex IV does not produce ROS [21] the function of the complex might have been protected.

Previous studies have shown that the rate of glycolysis increases in hyperoxia in isolated cloned AECII and in WI38 cells [24], [25]. However, the activities of important enzymes of glycolytic pathway such as glyceraldehyde-3-phosphate dehydrogenase, pyruvate kinase or phosphofructokinase did not decrease in hyperoxia [24], demonstrating that these sulfhydryl containing enzymes are not inactivated in hyperoxia. Studies have shown increased expression of hexokinase [26] and enhanced glucose utilization in A549 cells [8] and CHO cells in hyperoxia [27]. Although CHO cells were shown to remain months to years with 5-fold increase in glycolytic ATP production [27], severe growth impairment has been reported to occur in hyperoxia in glucose supplemented media [25]. Taken together, the precise effect of hyperoxia on glycolysis or glycolytic capacity remains inconclusive. Our current investigation shows that the rate of glycolysis did not change in hyperoxia compared to normoxia. However, maximal glycolytic capacity that could be achieved by MLE-12 cells in hyperoxia due to shifting of oxidative phosphorylation to glycolysis due to oligomycin treatment was decreased in hyperoxia compared to normoxia. This data show that MLE-12 cells could not overcome disruption of oxidative energy supply due to decreased glycolytic capacity of these cells. This could be an inherent characteristic for each cell type. Since glycolytic reserve is the difference between glycolytic capacity (maximal in presence of glucose + oligomycin) and glycolysis in presence of glucose, our data show the glycolytic reserve is decreased in hyperoxia as compared to normoxia in MLE-12 cells, suggesting that prolonged hyperoxia could cause cell death due to energy deficiency. In these conditions enhanced glucose utilization in hyperoxia could produce minimal energy for a limited time as previously reported, as the maximal glycolytic capacity of these cells are reached [8]. Our data show that there was a significant increase in the non-glycolytic acidification in hyperoxia compared to normoxia (Fig 3C). Since the cells were equilibriated in glutamine containing media for 1 hour in the micro-titer plate in a non-CO_2_ incubator after exposure to hyperoxia, we speculate that glutamine oxidation contributed to this increase in non-glycolytic ECAR via increase CO_2_ production in the mitochondria. It has been shown that glutamine can be used as an oxidizable substrate for ATP synthesis in cells under hyperoxic stress [28]. Since aconitase is inactivated in hyperoxia [29], pyruvate cannot be oxidized in the TCA cycle producing NADH. However, glutamine enters the TCA cycle after the aconitase step and therefore could be oxidized in the TCA cycle and produce limited amounts of NADH. Therefore, glutamine supplementation produces ATP under hyperoxia and allows cells to survive for a longer period of time [28]. Thus, the increase in non-glycolytic ECAR could be due to the enhanced oxidation of glutamine in hyperoxia.

In summary our study show that both glycolysis and oxidative phosphorylation are adversely affected by hyperoxia that could limit the energy supply for the metabolic function of lung cells in isolation or in the whole lung. Loss of energy supply could impair lung growth in premature infants subjected to hyperoxic ventilation. Additionally, lung repair following oxidative insult could also be adversely affected due to loss of energy availability.

## References

[1] Schober P, Schwarte LA (2012) From system to organ to cell: oxygenation and perfusion measurement in anesthesia and critical care. J Clin Monit Comput 26: 255–265. 22437884 10.1007/s10877-012-9350-4 PMC3391361

[2] Abman SH, Warady BA, Lum GM, Koops BL (1984) Systemic hypertension in infants with bronchopulmonary dysplasia. J Pediatr 104: 928–931. 6547171 10.1016/s0022-3476(84)80501-6

[3] Dos Santos CC (2007) Hyperoxic acute lung injury and ventilator-induced/associated lung injury: new insights into intracellular signaling pathways. Crit Care 11: 126. 17466082 10.1186/cc5733 PMC2206466

[4] Ratner V, Starkov A, Matsiukevich D, Polin RA, Ten VS (2009) Mitochondrial dysfunction contributes to alveolar developmental arrest in hyperoxia-exposed mice. Am J Respir Cell Mol Biol 40: 511–518. 19168698 10.1165/rcmb.2008-0341RC PMC3269235

[5] Brand MD, Nicholls DG (2011) Assessing mitochondrial dysfunction in cells. Biochem J 435: 297–312. 21726199 10.1042/BJ20110162 PMC3076726

[6] Sanders SP, Zweier JL, Kuppusamy P, Harrison SJ, Bassett DJ, et al (1993) Hyperoxic sheep pulmonary microvascular endothelial cells generate free radicals via mitochondrial electron transport. J Clin Invest 91: 46–52. 8380815 10.1172/JCI116198 PMC329993

[7] Guidot DM, McCord JM, Wright RM, Repine JE (1993) Absence of electron transport (Rho 0 state) restores growth of a manganese-superoxide dismutase-deficient Saccharomyces cerevisiae in hyperoxia. Evidence for electron transport as a major source of superoxide generation in vivo. J Biol Chem 268: 26699–26703. 8253804

[8] Allen CB, White CW (1998) Glucose modulates cell death due to normobaric hyperoxia by maintaining cellular ATP. Am J Physiol 274: L159–164. 9458814 10.1152/ajplung.1998.274.1.L159

[9] Scatena R, Messana I, Martorana GE, Gozzo ML, Lippa S, et al (2004) Mitochondrial damage and metabolic compensatory mechanisms induced by hyperoxia in the U-937 cell line. J Biochem Mol Biol 37: 454–459. 15469733 10.5483/bmbrep.2004.37.4.454

[10] Schoonen WG, Wanamarta AH, van der Klei-van Moorsel JM, Jakobs C, Joenje H (1990) Respiratory failure and stimulation of glycolysis in Chinese hamster ovary cells exposed to normobaric hyperoxia. J Biol Chem 265: 1118–1124. 2358458

[11] Rogers GW, Brand MD, Petrosyan S, Ashok D, Elorza AA, et al (2011) High throughput microplate respiratory measurements using minimal quantities of isolated mitochondria. PLoS One 6: e21746. 21799747 10.1371/journal.pone.0021746 PMC3143121

[12] Carney DN, Bunn PA Jr, Gazdar AF, Pagan JA, Minna JD (1981) Selective growth in serum-free hormone-supplemented medium of tumor cells obtained by biopsy from patients with small cell carcinoma of the lung. Proc Natl Acad Sci U S A 78: 3185–3189. 6265940 10.1073/pnas.78.5.3185 PMC319525

[13] Reiss OK (1966) Studies of lung metabolism. I. Isolation and properties of subcellular fractions from rabbit lung. J Cell Biol 30: 45–57. 4226009 10.1083/jcb.30.1.45 PMC2106992

[14] Dervartanian DV, Veeger C (1964) Studies on Succinate Dehydrogenase. I. Spectral Properties of the Purified Enzyme and Formation of Enzyme-Competitive Inhibitor Complexes. Biochim Biophys Acta 92: 233–247. 14249115

[15] De Duve C (1971) Tissue fractionation. Past and present. J Cell Biol 50: 20d–55d. 4327465

[16] Suarez MD, Revzin A, Narlock R, Kempner ES, Thompson DA, et al (1984) The functional and physical form of mammalian cytochrome c oxidase determined by gel filtration, radiation inactivation, and sedimentation equilibrium analysis. J Biol Chem 259: 13791–13799. 6094530

[17] Berry EA, Trumpower BL (1987) Simultaneous determination of hemes a, b, and c from pyridine hemochrome spectra. Anal Biochem 161: 1–15. 3578775 10.1016/0003-2697(87)90643-9

[18] Shenberger JS, Dixon PS (1999) Oxygen induces S-phase growth arrest and increases p53 and p21(WAF1/CIP1) expression in human bronchial smooth-muscle cells. Am J Respir Cell Mol Biol 21: 395–402. 10460757 10.1165/ajrcmb.21.3.3604

[19] McGrath-Morrow SA, Stahl J (2001) Growth arrest in A549 cells during hyperoxic stress is associated with decreased cyclin B1 and increased p21(Waf1/Cip1/Sdi1) levels. Biochim Biophys Acta 1538: 90–97. 11341986 10.1016/s0167-4889(00)00142-7

[20] Carlson DE, Pumplin DW, Ghavam S, Fiedler SM, Chiu WC, et al (2005) ATP accelerates respiration of mitochondria from rat lung and suppresses their release of hydrogen peroxide. J Bioenerg Biomembr 37: 327–338. 16341777 10.1007/s10863-005-8644-3

[21] Turrens JF (2003) Mitochondrial formation of reactive oxygen species. J Physiol 552: 335–344. 14561818 10.1113/jphysiol.2003.049478 PMC2343396

[22] Campian JL, Qian M, Gao X, Eaton JW (2004) Oxygen tolerance and coupling of mitochondrial electron transport. J Biol Chem 279: 46580–46587. 15328348 10.1074/jbc.M406685200

[23] Freeman BA, Crapo JD (1981) Hyperoxia increases oxygen radical production in rat lungs and lung mitochondria. J Biol Chem 256: 10986–10992. 7287745

[24] Simon LM, Raffin TA, Douglas WH, Theodore J, Robin ED (1979) Effects of high oxygen exposure on bioenergetics in isolated type II pneumocytes. J Appl Physiol 47: 98–103. 224021 10.1152/jappl.1979.47.1.98

[25] Balin AK, Goodman BP, Rasmussen H, Cristofalo VJ (1976) The effect of oxygen tension on the growth and metabolism of WI-38 cells. J Cell Physiol 89: 235–249. 972165 10.1002/jcp.1040890207

[26] Allen CB, Guo XL, White CW (1998) Changes in pulmonary expression of hexokinase and glucose transporter mRNAs in rats adapted to hyperoxia. Am J Physiol 274: L320–329. 9530166 10.1152/ajplung.1998.274.3.L320

[27] van der Valk P, Gille JJ, van der Plas LH, Jongkind JF, Verkerk A, et al (1988) Characterization of oxygen-tolerant Chinese hamster ovary cells. II. Energy metabolism and antioxidant status. Free Radic Biol Med 4: 345–356. 3384344 10.1016/0891-5849(88)90086-x

[28] Ahmad S, White CW, Chang LY, Schneider BK, Allen CB (2001) Glutamine protects mitochondrial structure and function in oxygen toxicity. Am J Physiol Lung Cell Mol Physiol 280: L779–791. 11238020 10.1152/ajplung.2001.280.4.L779

[29] Gardner PR, Nguyen DD, White CW (1994) Aconitase is a sensitive and critical target of oxygen poisoning in cultured mammalian cells and in rat lungs. Proc Natl Acad Sci U S A 91: 12248–12252. 7991614 10.1073/pnas.91.25.12248 PMC45414

